# Editorial

**Published:** 1840-02-22

**Authors:** 


THE MEDICAL EXAMINER.

PHILADELPHIA, FEBRUARY 22, 1840.

### PHYSICAL EXPLORATION.

In the last number of the Southern Medical and Surgical Journal, we observe an editorial article relative to the utility, practically speaking, of physical examinations.

The editor does by no means undervalue this mode of examination, but he lays unnecessary stress upon the difficulties of acquiring a knowledge of it. Hence he infers that the rational or functional signs must be relied upon by the practitioner who cannot acquire the troublesome art of physical examination.

Now, there is some truth in all this; but still, in the main, the editor is in the wrong. He evidently commits the ordinary mistake of supposing the physical signs and the natural symptoms to be antagonizing methods of diagnosis. This is an erroneous belief. The two methods are clearly connected; and it is a matter of every day observation, that the most thorough knowledge of the rational symptoms is generally connected with the greatest skill in physical signs. This, indeed, is a natural result; for a course of study which clears up the intricacies of a disease, extends its influence to the whole series of symptoms by which it is known. Hence the rational signs of thoracic diseases have assumed a new importance within a few years; and the diagnosis is not only facilitated by them when the physical signs would, of themselves, guide us to a correct result,—but they serve in many cases where the physical signs are insufficient, for the plain reason that the disease is but commencing. The greater certainty of the physical signs gives them value, not only as positive, but as negative proofs of the disease; hence we use them largely by the method of exclusion. That is, we decide that certain diseases do not exist, because their pathognomonic physical signs are absent,—and afterwards we resort to the rational symptoms, which acquire additional importance from our knowledge that the diseases which remain for our examination are but few in number.

As to the certainty of physical signs, it is almost unnecessary to speak. This is shown by every day experience; and if new evidence were requisite at this period, we need only refer to the daily observation of those who are in the habit of seeing these signs pointed out to them under circumstances which forbid the possibility of illusion. That the physical signs are not all-sufficient, is true; but, at the same time, they are much more accurate than any other known means of investigation. Even should the physician who resorts to them be but indifferently acquainted with them, the chances of error are not increased, but a mistake becomes more evident, for the vagueness of ordinary diagnosis does not shield it from observation. An opinion is, to a great extent, positive, and is shown to be true or false.

We are persuaded that even the difficulties of acquiring a knowledge of physical diagnosis, are in some degree exaggerated. It is true, a complete knowledge of them, and a facility in their application, are difficult, not only because time and opportunities are necessary to learn the signs themselves, but because they imply in their very nature an acquaintance with the history and pathology of pectoral diseases. Still, a knowledge which is quite sufficient for most purposes, and is a valuable aid to a physician who is obliged to rely mainly upon the rational symptoms, is neither very difficult, nor liable to lead to erroneous conclusions.

